# A comprehensive study of modified three-month pediatrics training curriculum at Shahid Beheshti University of Medical Sciences and its impact on student satisfaction

**DOI:** 10.1186/s12909-024-05408-z

**Published:** 2024-04-22

**Authors:** Amirhossein Hosseini, Mobina Fathi, Nahid Saadati, Koroush Vahidshahi, Mohammadmahdi Nasehi, Fereshteh Karbasian, Aliakbar Sayyari, Fatemeh Malek, Mahmoud Hajipour

**Affiliations:** 1 Pediatric Gastroenterology, Hepatology and Nutrition Research Center, Research Institute for Children’s Health, Shahid Beheshti University of Medical Sciences, Tehran, Iran; 2 Student Research Committee, School of Medicine, Shahid Beheshti University of Medical Sciences, Tehran, Iran; 3 Department of Pediatrics, School of Medicine, Shahid Beheshti University of Medical Sciences, Tehran, Iran; 4 Pediatric Cardiology, Faculty of Medicine, Shahid Modarres Hospital, Shahid Beheshti University of Medical Sciences, Tehran, Iran; 5 Pediatric Neurology Research Center, Research Institute for Children’s Health, Mofid Children’s Hospital, Shahid Beheshti University of Medical Sciences, Tehran, Iran; 6 Department of Pediatric Gastroenterology and Hepatology, Ali_Asghar Children’s hospital, Iran University of Medical Sciences, Tehran, Iran; 7 Pediatric Congenital Hematologic Disorders Research Center, Research Institute for Childern’s Health, Shahid Beheshti University of Medical Sciences, Tehran, Iran

**Keywords:** Pediatric course, Educational curriculum, Satisfaction, Medical student

## Abstract

**Objectives:**

Continuous curriculum improvements reveal the dedication of policy-makers to raising the quality of education and student learning. This study aims to report the impact of curriculum changes to the three-month pediatric course curriculum at Shahid Beheshti University of Medical Sciences (SBMU) on the satisfaction levels of medical students.

**Methods:**

One hundred eighteen 4^th^-5^th^ years medical students, who had completed their pediatric clinical rotation in SBMU-affiliated teaching hospitals including Mofid Children Hospital, Loghman Hakim Hospital, Shohada-e-Tajrish Hospital, and Imam Hossein Hospital from January to December 2022 were included in this cross-sectional study. After obtaining informed consent, a questionnaire was sent out to all participants, that included 27 statements about the impact of the modified curriculum on their satisfaction with their learning and performance. SPSS version 22 was used to analyze the data.

**Results:**

The level of satisfaction of trainees from attending clinics was 82-56%, prior introduction to the course was about 82%, and attending general hospitals (all hospitals except Mofid Children hospital, which is the only children hospital affiliated to SBMU) was 82-97%. The quality of patients-based learning was reported in terms of attendance at morning report sessions which was 92.3%, attendance at ward rounds, which was 71.8%, and attendance at clinics, which was 62.4%. The satisfaction rate from the senior attending mentor was 96.5%. The satisfaction rate of the pathology course was 67.2%, and the radiology was 82.4%. The satisfaction level of medical students from the infectious disease department was 70% and the gastroenterology department was 83.8%. The level of satisfaction with the implementation of the twelve-week program was 68.7%, with the expressiveness and usability of the presentation of materials was 53.9%, with the compatibility of the exams with the presented materials was 92%, and withholding weekly exams was 86.8%. The satisfaction rate of using the materials presented in the final exam in the digestive department and the infectious department was 85% and 68%, respectively. The overall satisfaction rate of the training course was 76.66%.

**Conclusion:**

The results provide vital insights for improving medical education. According to this study, medical student satisfaction with the pediatric curriculum after its recent revisions was in a satisfactory range. Attendance at clinics, information sharing, patient-based learning, practical training, attending mentorship, curriculum clarity, and alignment with student expectations all contributed to participants' high levels of satisfaction.

**Supplementary Information:**

The online version contains supplementary material available at 10.1186/s12909-024-05408-z.

## Introduction

Every educational system exists to achieve certain educational aims [1, 2]. It will be hard to move and activate precisely and eventually obtain that system's educational aims if the targeted goals are not appropriately settled and evaluated, and the priorities are not described and clarified vividly. Thus, based on the educational goals, curriculum design, evaluation, and intersystem educational activities are being planned [1]. Following the establishment of the Ministry of Health and Medical Education, the authorities and officials responsible for medical education have recognized the improvement of educational quality as a major priority, particularly in the context of not compromising patients' safety at educational hospitals [3].

Numerous measures have been taken by the individual universities to adjust the curricula, particularly in medical schools, to be consistent with those of worldwide standards. These changes include the implementation of an improved teaching and evaluation system. To ascertain whether the new methods are gaining superior results, a continuous evaluation system is necessary [4, 5]. The interconnection of the administrative structures of the medical school and the teaching hospitals, the increased responsibilities of instructors and administrators, and the complexity of the curriculum as a system of interconnected components result in significant impacts from every new alteration [5].

The clinical phases of continuing medical education (in Iran the clinical phase of medical education is presented in two phases, 2 years as medical students or externs and two years as medical interns) might be regarded as the most crucial ones since they allow students to translate their academic knowledge into a variety of clinical skills. However, unfortunately, medical students mostly highlight a higher level of dissatisfaction in these years, when compared to the preclinical stages [3]. Students' educational experiences and opinions about the course subject, organization, structure, and overall quality are unquestionably significant in determining the effectiveness of the curriculum. As a result, they can be considered a valuable resource in the process of curriculum formation and evaluation. In this regard, evaluating the opinions of the students regarding the acquisition of clinical skills can be one of the learning-facilitating activities in the clinical setting [6]. Thus, taking into account their perspectives is one of the techniques to evaluate educational methods, and clinical training systems, and subsequently improve educational quality [3].

The needs to regularly revise and update the materials and training content of pediatric clinical rotation is particularly important since pediatrics is one of the most major and important clinical rotations. The purpose of the current study was to determine how satisfied medical students were with the revisions made to the pediatric curriculum.

## Method

### Study design and ethical approval

This is a cross-sectional study approved by the Shahid Beheshti University of Medical Sciences Ethics Committee with the following approval code: SBMU.MSP.REC.1399.455. Participants provided their consent as necessary and were informed of the confidentiality of their information before data collection.

### Participants selection

The study population was made up of 4^th^-5^th^ years medical students who had completed their three-month pediatric rotation in 2022 at one of the affiliated children's hospitals of the Shahid Beheshti University of Medical Sciences, including Mofid Children's Hospital, Loghman Hakim Hospital, Imam Hussein Hospital, Shohada-e-Tajrish hospital, and Mahsih Daneshvari hospital. All students who were not available at the time of the survey or who declined to participate were excluded (zero participants were excluded).

### Participants' characteristics

The study was conducted on 118 medical students aged 22 to 24 years who had finished their three-month pediatric rotation at Mofid Children's Hospital, Loghman Hakim Hospital, Imam Hussein Hospital, and Shohada-e-Tajrish Hospital.

This section explores the findings of our study, which attempted to determine how satisfied medical students were with their pediatric rotation. We investigated some aspects of their experiences, including communication efficacy, engagement with clinical settings, satisfaction with mentors, alignment of covered materials with curriculum, and more, using thorough surveys and evaluations.

### Designing the questionnaire

We designed a questionnaire with multiple sections to evaluate the students’ satisfaction in several aspects. The questionnaire was made of 3 sections, including Satisfaction with Program Implementation, the Quality of Patient-Based Learning, and Satisfaction with Attendings and Mentors. 27 questions were asked in this questionnaire during the process (see Supplementary file).

### Data collection and analysis

The researchers developed checklists that covered components crucial to the study's objectives. These checklists were delivered in person to the participating medical students. Student satisfaction with program introduction, class scheduling, materials, study tactics, study tools, exams and evaluations, and the role of preceptors were only a few of the program execution-related areas covered by the collected data. Information was also gathered from medical students who could not be reached at the time of the in-person survey through phone calls.

### Implementation phases

#### Assessment and problem identification

The difficulties faced throughout the program were discussed in depth during several meetings with medical students. Students expressed concerns about a range of issues, including how poorly teaching subjects corresponded with the curriculum, how much information was covered on final examinations, and how little they were exposed to certain clinical departments. Pediatric attendings and mentors were also asked for their opinions on the program, as well as their experiences and observations. To classify and handle the identified difficulties, a group of professionals got together. They included department chiefs, educational deputies, and pediatric attendings.

The noted issues were categorized as follows:

i. Theoretical subjects that are mentioned in the curriculum are not covered during the rotations.
ii. Students only get a small amount of exposure to certain departments.
iii. The presence of students in the pediatric emergency wars and general pediatrics departments was scant.
iv. The materials for final exams were inappropriately covered.
v. Patient selection for the morning report.
vi. The size of the students groups for each clinical rotation and the scarcity of attendings and mentors.
vii. The lack of digital resources and restricted access of students to these materials.
viii. Theoretical class sessions are scheduled at inconvenient times, requiring students to abandon clinical departments to attend afternoon classes.

#### Intervention and process enhancement

Interventions were created to address the issues and improve the educational program based on the problem classification and expert panel talks. The next actions were taken:

i. *Curriculum Alignment:* The curriculum was updated to include all of the Ministry of Health's essential subjects, as well as new subjects pertinent to clinical practice.
ii. *Faculty Involvement:* It was coordinated with faculty members to make sure that teaching subjects were thoroughly covered and that the content of the lessons and the sources used for the educational course were in line.
iii. *Didactic Sessions:* The didactic sessions were structured based on head theme panels (for example, gastrointestinal, and respiratory), scheduled to occur concurrently with departmental meetings, and planned at the beginning of the 12-week course.
iv. *Clinical Rotations:* A patient load of each ward was taken into consideration when allocating clinical rotations. General pediatrics would receive 3 weeks, gastrointestinal and nephrology would receive 2, and others would receive 1.
v. *Exam format:* New sections with thorough coverage of distinct thematic subjects were added to the test format. The examination room at the university used a computer-based system for exams.
vi. *Morning Reports:* The Mofid Children's Hospital first implemented student-based Morning Report Sessions. These meetings took place concurrently with the hospital's morning report, and they were led by the attendings.
vii. *Student Group Division:* To encourage group discussions, students were divided into smaller groups.
viii. *Resource Accessibility:* For unrestricted student access and download, audio lecture slides have been recorded and posted to the pediatric department's website.

#### Adopting the revisions to the curriculum

The shortcomings of the educational program were addressed and modifications were adopted to improve the entire educational experience for medical students through systematic assessment, identification of difficulties, and targeted interventions. These actions intended to establish a more thorough and balanced learning environment, harmonize the curriculum, and expand exposure to other clinical areas.

## Results

### Variability in student satisfaction across different clinics

**Fig. 1:**
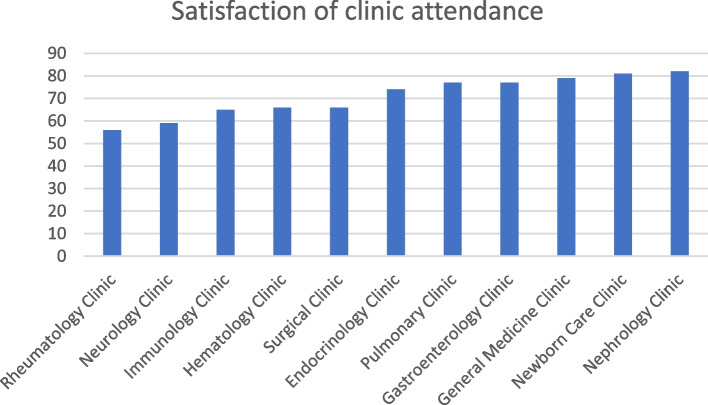
The satisfaction of medical students in clinics

During their clinical training phase, student satisfaction was examined across various specialist clinics, revealing a wide range of experiences. The evaluation covered a variety of clinics, each linked to a particular medical specialty. Intriguing differences and new information about the student experience in several clinical settings were revealed by the resulting satisfaction rates (Fig. 1, Table 1):

- Rheumatology Clinic: Students expressed a satisfaction rate of 56%, indicating a moderate level of contentment with their experiences in this clinic.
- Neurology Clinic: Within the Neurology Clinic, students reported a satisfaction rate of 59%, suggesting a similar moderate level of satisfaction.
- Immunology Clinic: Satisfaction levels within the Immunology Clinic stood at 65%, indicating a relatively higher degree of contentment among students.
- Hematology Clinic: Observations from the Hematology Clinic revealed a satisfaction rate of 66%, signaling a balanced satisfaction level among students.
- Surgical Clinic: Students in the Surgical Clinic displayed a satisfaction rate of 66%, aligning closely with the satisfaction reported in other clinics.
- Endocrinology Clinic: Satisfaction among students within the Endocrinology Clinic reached 74%, reflecting a noteworthy level of contentment.
- Pulmonary Clinic: The Pulmonary Clinic garnered a satisfaction rate of 77%, suggesting an elevated level of satisfaction among participating students.
- Gastroenterology Clinic: Students in the Gastroenterology Clinic indicated a satisfaction rate of 77%, mirroring the satisfaction observed in the Pulmonary Clinic.
- General Medicine Clinic: Notably, the General Medicine Clinic yielded a satisfaction rate of 79%, reflecting a relatively high level of students’ contentment.
- Newborn Care Clinic: Students' satisfaction levels reached 81% within the Newborn Care Clinic, portraying a robust sense of contentment.
- Nephrology Clinic: The Nephrology Clinic emerged with the highest reported satisfaction rate of 82%, underscoring an exceptional level of student satisfaction in this clinical domain.

**Table 1:** Student satisfaction across specialized clinics

**Clinic**	**Satisfaction Rate (%)**
Rheumatology Clinic	56
Neurology Clinic	59
Immunology Clinic	65
Hematology Clinic	66
Surgical Clinic	66
Endocrinology Clinic	74
Pulmonary Clinic	77
Gastroenterology Clinic	77
General Medicine Clinic	79
Newborn Care Clinic	81
Nephrology Clinic	82

### Course introduction and curriculum satisfaction

*Laying the foundation:* The degree of participant satisfaction was noticeably high at the start of the program. Their satisfaction with the initial program introduction and communication was clear. 94% of respondents said they were satisfied with how well the course contents, study techniques, and reference resources were explained. Furthermore, a commendable 82.1% of people were satisfied with how exam specifics were delivered (Table 2).

**Table 2:** Evaluation of education and patient interaction in clinical settings

**Aspect**	**Satisfaction Rate (%)**
Gastroenterology Department	
- Use of patient cases for presentations	76.5
- Alignment of presentation titles	82
Infectious Diseases Department	
- Use of patient cases for presentations	70.8
- Alignment of presentation titles	68.2
Student Satisfaction with Mentors	
- Professionalism	95.2
- Presence during clinical interactions	94.6
- Problem-solving capacity	94.9
Student Satisfaction with Senior Mentors	
- Professional behavior	96.5
- Involvement	92.7
- Problem-solving capacity	94.6
Pathology Instruction Satisfaction	
- Course related to clinical programs	67.2
- Faculty presence	74.2
- Assessment strategies	68.1
- Use of reports	55.4
Radiology Instruction Satisfaction	
- Lesson alignment	82.4
- Teacher attendance	88.4
- Evaluation techniques	80.3
- Report utilization	81.9
Infectious Diseases Department Satisfaction	
- Program introduction	70
- Complete weekly program presentations	62.8
- Staff readiness for student integration	65.7
- Staff interactions	70.6
- Importance of fellowships	64.5
- Instructor awareness of student presence	72.2
Program Execution and Satisfaction	
- Low satisfaction	1.7
- Moderate satisfaction	29.6
- High satisfaction	68.7
Instructor Alignment and Awareness	
- Strong alignment	78.8
- Moderate alignment	17.6
- Low alignment	2.7
- Very aware of program changes	77.4
- Somewhat aware of program changes	18.3
- Not at all aware of program changes	4.3
Effectiveness of Presentation Materials	
- High satisfaction	53.9
- Moderate satisfaction	40.9
- Low satisfaction	5.2
Teaching Schedule Congruence	
- Good schedule alignment	71.1
- Moderate schedule alignment	28.9
- Low schedule alignment	0
Examination Satisfaction	
- Alignment with material	92
- Administration of weekly exams	86.8
End-of-Section Examination Satisfaction	
- Gastroenterology department	85
- Infectious Diseases section	68
Overall Course Satisfaction	76.66

### Comparison of student satisfaction in different hospitals

The findings show various tendencies among the hospitals. Loghman Hakim Hospital demonstrated strong faculty involvement in student instruction (97.2%) and awareness (96.3%). Students expressed high levels of satisfaction (95.9%) with contacts with staff, and the hospital showed a high level of preparation for student integration (97%). Shohada-e-Tajrish Hospital showed comparable encouraging trends, with considerable faculty involvement in student instruction (96.06%) and awareness (97.19%). The hospital's readiness for student integration was slightly lower (92.4%) even though student satisfaction with staff interactions remained high (93.03%). Imam Hossein Hospital demonstrated good faculty involvement in student education (90.29%) and faculty awareness (94.86%). The readiness for student integration (83.4%) and student satisfaction with staff interactions (82%) both have space for development (Table 2).

### Evaluation of patient interaction during the training period

- *Student engagement in morning report sessions:* A noteworthy number of students (92.3%) reported attending more than 10 morning report sessions, demonstrating a strong dedication to the educational process. A small minority (1.7%) reported participation in fewer than 5 sessions, whereas a substantial amount (6%) highlighted their presence in 5 to 10 meetings. This information highlights the importance of students' active participation in the learning environment of morning report talks (Table 2).
- *Student presence in ward rounds:* Examining the presence of students during ward rounds revealed some interesting patterns. The vast majority of students (71.8%) participated in more than seven ward rounds, demonstrating their commitment to practical clinical experience. A sizable amount (24.8%) attended 4 to 7 rounds, whereas a far lower percentage (3.4%) joined fewer than 4. These findings show the range of clinical encounters that the students had as well as how they struck a balance between active participation and the demands of their training schedule (Table 2).
- *Student presence in outpatient clinics:* In more than 20 outpatient clinic visits, a sizable percentage (62.4%) of students dealt with patients, demonstrating a remarkable exposure to a variety of medical conditions. Furthermore, a sizable portion (36.8%) attended ten to twenty clinics, whereas a negligible portion (0.9%) attended less than ten. These results highlight the extent of the student's involvement with outpatient settings and their growing competency in dealing with patients (Table 2).

### Evaluation of education and patient interaction in clinical settings

**Fig. 2:**
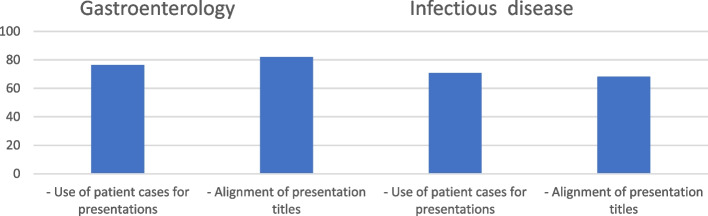
The satisfaction of medical students in gastroenterology and infectious disease wards

- *Gastroenterology department: *According to the survey, 76.5% of students used patient cases for presentations in the gastroenterology wards, illustrating the close relationship between academic learning and practical application. Additionally, an optimistic 82% of respondents indicated that the presentation titles and the department's patient group were in line, highlighting the applicability of education to real-world situations (Fig. 2).
- *Infectious diseases department:* Similar results were shown in the Department of Infectious Diseases, where 70.8% of students included patient cases in their presentations, demonstrating how real-world cases are incorporated into the classroom. The efforts to link curricula with clinical experiences are highlighted by the 68.2% alignment of presentation titles with the patient context (Fig. 1).
- *Student satisfaction with preceptors:* Mentors were highly rated by students for their professionalism (95.2%), presence during clinical interactions (94.6%), and capacity to handle problems (94.9%). These results highlight how beneficial preceptors and mentors are for the educational process (Table 2).
- *Student satisfaction with senior preceptors:* Similar levels of satisfaction were reported with senior preceptors, with 96.5% expressing satisfaction with their professional behavior, 92.7% with their involvement, and 94.6% with their ability to resolve problems. This emphasizes the critical function of knowledgeable mentors in creating a supportive learning environment (Table 2).
- *Pathology course satisfaction:* Student satisfaction in the course of pathology varied. While 67.2% of respondents were satisfied with how courses were related to clinical programs, 74.2% were satisfied with faculty presence, and 68.1% were satisfied with assessment strategies, only 55.4% of respondents said they were satisfied with how reports were used, which suggests room for improvement (Table 2).
- *Radiology course satisfaction:* Satisfaction ratings were comparatively greater in radiology courses. Material alignment was rated as satisfactory by 82.4% of respondents, faculty attendance by 88.4%, evaluation techniques by 80.3%, and report utilization by 81.9% of respondents. These findings show that the radiology course has been successfully incorporated into the curriculum (Table 2).
- *Infectious diseases department satisfaction:* In the department of infectious diseases, satisfaction with program introduction was at 70%, complete weekly program presentations were at 62.8%, staff readiness for student integration was at 65.7%, staff interactions were at 70.6%, and the role of clinical. fellowships in student education were at 64.5%, and instructors' awareness of student presence and programs was at 72.2%. These results highlight many facets of departmental satisfaction (Table 2).
- *Program execution and satisfaction:* The outcomes show a high overall degree of satisfaction with the twelve-week course. Only 1.7% of respondents reported low satisfaction, a small minority (29.6%) moderate, and a sizable majority (68.7%) high satisfaction. This shows that the execution and content of the curriculum have been well received (Table 2).
- *Instructor alignment and awareness:* When it came to alignment with specific instructors, the majority (78.8%) rated strong alignment, followed by moderate alignment (17.6%), and low alignment (2.7%). 77.4% of respondents said they were well aware of program changes, 18.3% said they were somewhat aware, and 4.3% said they were not at all informed. These results underline the need for instructor transparency and communication about curriculum changes (Table 2).
- *Effectiveness of presentation materials:* Analyzing the present materials revealed several viewpoints. While 53.9% expressed a high level of satisfaction with the materials' applicability and relevance, 40.9% reported a moderate level of satisfaction, and 5.2% a lower level. This shows that there is space to increase the thoroughness and impact of educational materials (Table 2).
- *Teaching schedule congruence:* 71.1% of teachers reported good schedule alignment with the allocated hours, while 28.9% reported moderate alignment. Notably, no participants indicated low alignment, suggesting that teaching schedules and program requirements are frequently well-matched (Table 2).
- *Examination satisfaction: *Exam participant satisfaction also revealed good outcomes. An amazing 92% of respondents expressed satisfaction with how well tests were aligned with the presented materials. Additionally, 86.8% of respondents said they were satisfied with how the weekly exams were administered, indicating that the assessment strategies are usually well-liked (Table 2).
- *End-of-section examination satisfaction: *The Gastroenterology department scored an 85% satisfaction rating for end-of-section exams, whereas the Infectious Diseases section received a 68% rating. The different effects of evaluation procedures across various sections are highlighted here (Table 2).
- *Overall satisfaction:* The study's findings show a 76.66% total satisfaction rate. Despite differences in certain aspects, the participants' overall satisfaction with the educational experience is highlighted by this cumulative measure (Table 2).

## Discussion

The study's findings provided insight into how medical students and trainees saw and felt about participating in our revised twelve-week clinical rotation in the pediatric wards among educational hospitals affiliated to SBMU. The majority of participants expressed a favorable propensity for taking part in this clinical rotation, showing a high degree of satisfaction. This satisfaction was extended to their encounters with patients inside a professional environment as well as the caliber of training they had received. These findings have important career-related ramifications for medical professionals, affecting both their working environments and their contacts with other healthcare workers. Creating a good work atmosphere while in training can help practitioners become well-rounded professionals who not only have the medical knowledge needed to provide quality patient care but also the requisite interpersonal skills.

The study's attention to the patient group, which primarily seeks treatment in outpatient clinics, is significant. Students and trainees are exposed to a variety of health disorders due to the prevalence and range of diseases seen in these clinics, which enables them to connect their academic learning with the community's predominant health challenges. It should be noted, though, that some rare cases, frequently chronic and uncommon, remain outside the purview of these clinics, which could result in a bias in the types of cases seen.

The result is consistent with other studies, especially in pediatric departments, where outpatient clinics act as active learning environments because of their duration and the built-in patient-physician connections [7, 8]. In these clinics, the benefit of face-to-face patient engagement makes clinical examinations, patient conversations, and the sharing of ideas easier.

The study's findings also demonstrate how crucial it is to plan for and prioritize medical education in a variety of clinical settings, especially outpatient clinics. By considering a larger spectrum of the community's prevalent health challenges, such planning should encourage a well-rounded medical education. Encouraging trainees to take part in these activities in collaboration with other healthcare units could lead to a deeper understanding of patient assessment, case management, complaint reporting, and follow-up. It is important to be aware of the limitations of this study, including potential biases in self-reported data and the focus on a specific healthcare environment. The conclusions drawn from the data are also dependent on the writers' perceptions and experiences, which can fluctuate based on the situation. The study does, however, emphasize the significance of practical experience in assessing the readiness and competency of prospective medical professionals.

Because every type of planning to improve clinical training quality depends on the identification of problems, inadequacies, and deficiencies existing in the educational system based on the target group's perspective, the current study sought to ascertain medical students' satisfaction with clinical training in the teaching hospitals in Tehran. The results showed that most participants were satisfied with clinical training techniques, clinical competence levels of faculties, and clinical training quality. They also voiced their displeasure with the clinical training goals, clinical evaluation procedures, clinical training tools and resources, and students' clinical competence levels. In this regard, Masic's research in Bosnia suggested that the students were dissatisfied with the issues that were present in their clinical skills training [9]. Only 28.4% of the students in the study by Jalili et al. were overall happy with the instruction they had received [10]. However, Guarino et al. also showed that even if there is room for improvement, overall student satisfaction with teaching is excellent [11].

## Conclusion

The study underlines the importance of improving the medical education curriculum. In addition to offering opportunities for skill development, these environments promote awareness of the demands for cooperative healthcare efforts and community health needs. Taking note of the lessons acquired from this research by medical education planners and policymakers to help create a more efficient and flexible healthcare workforce that is better prepared to address the issues posed by diverse patient populations and changing healthcare systems.

### Supplementary Information

**Supplementary Material 1.**

## Data Availability

The datasets used and/or analyzed during the current study are available from the corresponding author on reasonable request. Informed consent was obtained from all subjects and/or their legal guardian(s).
