# Supplementary material for: A comprehensive study of modified three-month pediatrics training curriculum at Shahid Beheshti University of Medical Sciences and its impact on student satisfaction
Source: BMC Med Educ. 2024 Apr 22;24:433. doi: 10.1186/s12909-024-05408-z (PMC11036761; doi:10.1186/s12909-024-05408-z)
Supplement: Supplementary file 1 — Supplementary Material 1. [file 12909_2024_5408_MOESM1_ESM.docx]

**Questionnaire:**

**Section A - Program Implementation Level**

| **Question** | **Score (0 to 10)** |
| --- | --- |
| 1. How well was the program introduction conducted at the beginning of the course? |  |
| 2. To what extent were program titles in semiology, instructors' names, and program locations presented to you? |  |
| 3. Were teaching content, study methods, and reference materials provided to you? | Yes □ No □ |
| 4. Were you informed about the exam method and schedule at the beginning of the course? | Yes □ No □ |
| 5. Any specific comments about program implementation? |  |

**Section B - Utilization of Patients During Training**

| **Question** | **Score (0 to 10)** |
| --- | --- |
| 1. How many morning reports were held during the course? |  |
| 2. How many ward rounds took place during the course? |  |
| 3. How many outpatient clinics were conducted during the course? |  |
| 4. Rate your satisfaction with various hospital departments: |  |
| - Infectious Diseases □ - Gastroenterology □ - Neurology □- Rheumatology □ - Endocrinology □ - Surgery □- Nephrology □ - Immunology □ - Hematology □  - General □ - Neonatology □ |  |
|  |  |
|  |  |
|  |  |
| 5. How well were topics coordinated with respective patient departments? |  |
| 6. To what extent were patients in the department used for lectures? |  |
| 7. Any comments about the educational benefits of different sections in this category? |  |

**Section C - Mentors**

| **Question** | **Score (0 to 10)** |
| --- | --- |
| 1. Rate your satisfaction with the professional behavior, ethics, and teaching approach of the mentor. |  |
| 2. Was the mnetor's presence and interaction appropriate? |  |
| 3. To what extent did the mentor address problems, if they existed? |  |
| 4. Rate your satisfaction with the professional behavior, ethics, and teaching approach of the chief mentor. |  |
| 5. Was the chief mentor's presence and interaction appropriate? |  |
| 6. To what extent did the chief mentor address problems, if they existed? |  |
| 7. Any comments about the mentor? |  |

**Section D - Satisfaction with Department (Personnel, Residents, Attendings)**

| **Question** | **Score (0 to 10)** |
| --- | --- |
| 1. How well was the department introduction program conducted at the beginning? |  |
| 2. How well was the weekly program presented, including instructor names and program locations? |  |
| 3. How prepared were department personnel to accept students? |  |
| 4. How appropriate was the interaction of department personnel with you? |  |
| 5. To what extent did residents contribute to your education? |  |
| 6. To what extent did fellows contribute to your education? |  |
| 7. How informed were instructors about your presence and program? |  |
| 8. Any comments about instructors, personnel, residents, fellows? |  |
